# *Enterococcus faecium* Stimulates Human Neutrophils via the Formyl-Peptide Receptor 2

**DOI:** 10.1371/journal.pone.0039910

**Published:** 2012-06-29

**Authors:** Dominik Alexander Bloes, Michael Otto, Andreas Peschel, Dorothee Kretschmer

**Affiliations:** 1 Interfaculty Institute of Microbiology and Infection Medicine, Cellular and Molecular Microbiology Section, University of Tübingen, Tübingen, Germany; 2 Pathogen Molecular Genetics Section, Laboratory of Human Bacterial Pathogenesis, National Institute of Allergy and Infectious Diseases, U.S. National Institutes of Health, Bethesda, Maryland, United States of America; Columbia University, United States of America

## Abstract

The human formyl-peptide receptor 2 (FPR2/ALX) senses phenol-soluble modulin (PSM) peptide toxins produced by pathogenic staphylococcal species and plays a crucial role in directing neutrophil influx during staphylococcal infection. However, it has remained unclear if FPR2 responds also to molecules from other bacterial pathogens. Here we analyzed a variety of Gram-positive and Gram-negative pathogens and found that apart from staphylococci only certain enterococcal strains have the capacity to stimulate FPR2/ALX. Most of the analyzed *Enterococcus faecium* but only sporadic *Enterococcus faecalis* strains released FPR2/ALX-stimulating molecules leading to neutrophil calcium ion fluxes, chemotaxis, and complement receptor upregulation. Among ten test strains vancomycin-resistant *E. faecium* had a significantly higher capacity to stimulate FPR2/ALX than vancomycin-susceptible strains, suggesting an association of strong FPR2/ALX activation with health-care associated strains. The enterococcal FPR2/ALX agonists were found to be peptides or proteins, which appear, however, to be unrelated to staphylococcal PSMs in sequence and physicochemical properties. Enterococci are among the most frequent invasive bacterial pathogens but the basis of enterococcal virulence and immune activation has remained incompletely understood. Our study indicates that previously unrecognized proteinaceous agonists contribute to *Enterococcus*-host interaction and underscores the importance of FPR2/ALX in host defense against major endogenous bacterial pathogens.

## Introduction

Mammalian immune cells have the capacity to detect secreted or surface-attached bacterial molecules, which are called pathogen-associated molecular patterns (PAMPs). Upon activation by PAMPs, the host responds with inflammation and activation of the immune system. However, most PAMPs do not represent virulence factors and are produced by both pathogens and commensals [1]. Formylated peptides are released by all kinds of bacteria and are sensed by the formyl-peptide receptor 1 (FPR1), a G-protein coupled receptor on mammalian leukocytes [2], [3]. Formylated peptides elicit neutrophil influx via FPR1 *in vivo* and activate neutrophils to release IL-8 and upregulate the complement receptor CD11b [4].

We have recently demonstrated that the FPR1-related formyl-peptide receptor 2 (FPR2/ALX), which responds only weakly to formylated peptides [4], is strongly activated by *S. aureus* peptide toxins named phenol-soluble modulins (PSMs) [5]. PSMs have cytolytic properties at micromolar concentrations and represent crucial virulence factors in highly virulent *S. aureus* lineages such as the community-associated methicillin-resistant *S. aureus* (CA-MRSA) USA300 and USA400 [6]. PSMs have also been shown *in vitro* and *in vivo* to contribute substantially to recruiting and activating neutrophils in CA-MRSA infections [6]. Of note, *S. aureus* also secretes the FLIPr protein, a highly specific inhibitor of human FPR2/ALX [7], which supports the notion that PSM-FPR2/ALX interactions are crucial in *S. aureus* infections and can be modulated by the pathogen. The extent of PSM release and corresponding FPR2/ALX-mediated neutrophil responses has recently been shown to be closely related to the virulence potential of staphylococci. Most pathogenic staphylococcal species bear *psm* genes in their genomes and produce PSMs, while both are usually absent from commensal species [8]. These findings indicate that FPR2/ALX is crucial in staphylococcal infections and may instruct the innate immune system about the virulence of an invading clone. However, it has remained unclear if FPR2/ALX responds only to staphylococcal pathogens or whether it may have a broader role in bacterial infections.

*Enterococcus faecium* and *Enterococcus faecalis* are colonizers of the human intestine and frequently cause opportunistic infections [9]. The two species seem to encode only a limited set of virulence factors; the mechanisms governing enterococcal pathogenicity have remained incompletely understood [9]. The fast resistance gain has made *E. faecium* an almost equally frequent cause of infection as *E. faecalis* [10], [11]. For the latter, a cytolysin and surface-associated aggregation substance proteins encoded on the so-called sex pheromone plasmids contribute to the induction of endocarditis in animal infection models [12], [13], [14]. Plasmid-encoded pheromone peptides direct transformation competence and some of these peptides, although lacking N-terminal formyl groups, have been shown to activate neutrophils via FPR1 [15], [16], [17]. Notably, it has remained unclear if enterococci or other human pathogens besides staphylococci release agonists of the FPR2/ALX receptor.

In an attempt to explore if FPR2/ALX is specific to staphylococcal bacterial infections or responds also to other microbes, we compared the capacities of bacterial pathogens to activate FPR2/ALX. Among several tested Gram-positive and Gram-negative species, only enterococcal isolates activated FPR2/ALX. This property was frequent among *E. faecium* but only rarely found in *E. faecalis* isolates. Interestingly, it was significantly associated with vancomycin resistance. The *E. faecium* FPR2/ALX agonists turned out to be of proteinaceous nature and to be probably unrelated to PSMs in terms of sequence, chromatographic behavior, synergistic haemolysis.

## Materials and Methods

### Bacterial strains, growth, and culture filtrates

Bacterial strains (Table 1) were grown at 37°C in tryptic soy broth (TSB) (AppliChem) for 17 h under agitation. Bacterial culture filtrates were obtained by centrifugation (10 min, 4°C at 5000× *g*) of overnight cultures grown in TSB and filtered through 0.2-µm pore size filters. Sterility of the samples was confirmed by plating aliquots on TSB agar plates. Enterococcal culture filtrates to be compared in a given experiment were standardized to the same optical density. Clinical isolates were identified to the species level using a Vitec 2 system (bioMerieux) and vancomycin resistance was detected by Vitec 2 or ellipsometry test (AB Biodisc) as described recently [18]. The identity of some isolates was also confirmed via matrix-assisted laser desorption/ionization time-of-flight mass spectrometry analysis (Axima Assurance, Shimadzu).

**Table 1:** Bacterial strains.

Strains	Description
*Enterocococcus faecalis:*	
BK4752, BK4737, BK4714, BK4684	vancomycin-susceptible, isolated from human blood cultures^a^
ST4138	vancomycin-susceptible, isolated from human feces^a^
VRE366, V583	vancomycin-resistant, isolated from human feces^a^; [40]
*Enterococcus faecium:*	
BK4705, BK4689, AN4639, BK2241	vancomycin-susceptible, isolated from human blood cultures^a^
BK526, BK474, BK463	vancomycin-resistant, isolated from human blood cultures^a^
ST4144	vancomycin-sensitive, isolated from human feces^a^
VRE517	vancomycin-resistant, isolated from human feces^a^
VRE392	vancomycin-resistant a
*Escherichia coli* BK2324	isolated from human blood culture a
*Staphylococcus aureus* USA300 LAC	CA-MRSA [41]
*Staphylococcus aureus* USA400 MW2	CA-MRSA [42]
*Streptococcus agalactiae* SK43	[43] a
*Yersinia enterocolitica* WA-314	Serotype O:8 [44] a
*Yersinia pseudotuberculosis* O1b	Serotype O1b [45] a

a From the strain collection of the diagnostics unit of the Medical Microbiology and Hygiene department, University of Tübingen.

### Measurement of calcium ion fluxes in HL60 cells and neutrophils

Recently described HL60 cells stably transfected with human FPR1 and FPR2/ALX [19], [20] were grown in RPMI medium (Biochrom) supplemented with 10% FCS (Sigma-Aldrich), 20 mM HEPES (Biochrom), penicillin (100 units ml^−1^), streptomycin (100 µg ml^−1^, GIBCO), and 1× Glutamax (GIBCO). Transfected cells were grown in the presence of G418 (final concentration 1 mg ml^−1^, Biochrom). Human neutrophils were isolated from blood of healthy volunteers by standard Histopaque/Ficoll gradient centrifugation [21]. To monitor calcium ion fluxes, neutrophils were loaded with the calcium-sensitive dye Fluo-3-AM (Molecular Probes) as described recently [5]. Neutrophils (1×10^6^ cells ml^−1^) were challenged with bacterial culture filtrates or synthetic FPR1 or FPR2/ALX ligand peptides. for 15 s and diluted in RPMI medium containing 0.05% human serum albumin (HSA). As positive controls, the synthetic peptides fMLF (20 nM; Sigma-Aldrich) or MMK1 (50 nM; LESIFRSLLFRVM-NH2, synthesized by EMC Microcollections), which are known to be highly specific agonists for FPR1 [22], [23], [24] or FPR2/ALX [5], [23], [24], [25], respectively, were used. Thereafter, calcium influx into the cytoplasm was measured via flow cytometry with a FACSCalibur (BectonDickinson). In order to block FPR2/ALX-dependent calcium influx, the *S. aureus*-derived specific inhibitor FLIPr was used. This molecule is known to specifically inhibit FPR2/ALX with only little inhibitory activity towards the related FPR1 [5], [7], [26]. Neutrophils were pre-incubated with FLIPr at a final concentration of 1 µg ml^−1^, for 20 min at room temperature under agitation.

### Neutrophil chemotaxis

Chemotaxis of neutrophils towards bacterial culture filtrates or synthetic peptides was determined by using fluorescence-labelled neutrophils that migrated through a 3-µm pore size polycarbonate trans-well filter as described recently [21]. Culture filtrates or synthetic peptides were applied as stimuli at the indicated concentrations. To measure the influence of FLIPr, cells were pre-incubated with FLIPr at final concentrations of 10 µg ml^−1^ for 20 min at room temperature under agitation. The relative fluorescence was measured, subtracted for the buffer control, and given as percentage of the positive control (25 nM MMK1).

### CD11b expression on human neutrophils

To test whether enterococcal culture filtrates promote upregulation of CD11b, 1×10^6^ neutrophils per well were applied to 100 µl RPMI with 0.05% HSA in 96 well round-bottom plates and 100 µl of culture filtrates were added to achieve a final concentration of 3% culture filtrate. After incubation for 60 min at 37°C, stimulation was stopped by centrifugation (8 min, 4°C at 350× *g*). Then, neutrophils were washed with ice-cold Dulbecco's Phosphate-Buffered Saline (DPBS; PAA Laboratories) (7 min at 350× *g*), anti-CD11b-PE or IgG-PE isotype control (1∶40 dilution; BD Pharmingen) were added, and samples were incubated for 30 min at 4°C in the dark. Afterwards, cells were washed with 200 µl ice-cold DPBS again and the pellets were resuspended in 300 µl DPBS. The samples were analyzed via flow cytometry with a FACSCalibur. For elucidating the inhibitory influence of FLIPr, PMN were incubated for 20 min at room temperature with 1 µg ml^−1^ final concentration of FLIPr. In order to inactivate any LPS trace contamination polymyxin B (Sigma-Aldrich) was added at a final concentration of 10 µg ml^−1^.

### Detection of PSM peptides by reversed-phase high-pressure liquid chromatography/electrospray mass spectrometry (RP-HPLC/ESI-MS)

The search for PSM peptides in enterococcal supernatants was performed using RP-HPLC/ESI-MS as described previously [8]. To that end, enterococcal culture filtrates from 8 h and 24 h of growth in shaken TSB cultures were applied and compared with culture filtrate of *S. aureus* USA300 (strain LAC) grown under the same conditions.

### Proteolytic stability of calcium ion flux inducers

To elucidate if FPR2/ALX-activating compounds in *E. faecium* supernatants are of proteinaceous nature, culture filtrates or the FPR2/ALX-specific agonist MMK1 were treated with an unspecific proteinase as described recently [21]. We used 1 unit ml^−1^ of proteinase K from *Tritirachium album* (immobilized on Eupergit® C; Sigma-Aldrich) to incubate the samples for 1 h at 37°C. Proteinase K Eupergit® C beads were subsequently removed by centrifugation at low speed (10 min at 250× *g*). In order to elucidate whether potential ligands were in fact degraded by proteinase K control samples were treated with beads, which had been boiled for 1 h at 99°C prior to incubation with culture filtrates. Proteolytically digested culture filtrates were used in the calcium flux assay with FPR2/ALX-transfected HL60 cells described above.

### Statistical methods

All statistical analyzes in this study were performed using Prism 5.04 software (GraphPad). Differences were analyzed for significance with the two-tailed Student's t-test or one-way ANOVA with Bonferroni's post test as indicated.

## Results

### FPR2/ALX does not respond to non-staphylococcal pathogens except *E. faecium*

**Figure 1:**
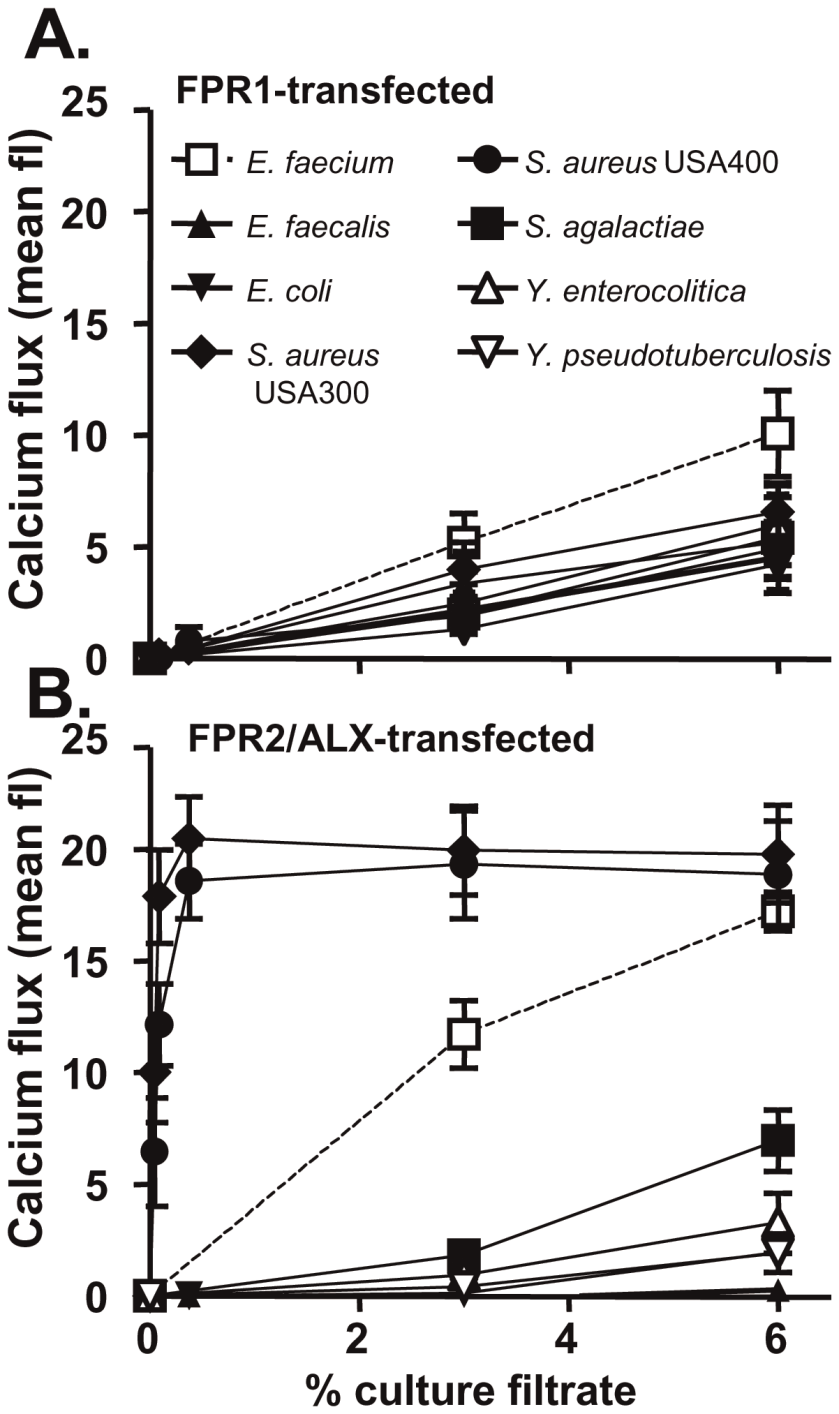
Induction of calcium ion flux in FPR1-transfected (A) or FPR2/ALX-transfected (B) HL60 cells. Data represent means ± SEM of 3 independent experiments with 3 different culture filtrates.

Our previous study demonstrated a correlation of virulence potential with the capacity to activate FPR2/ALX among staphylococci, but it has remained unclear if this applies only to staphylococcal or to further bacterial pathogens [8]. In order to address this question, we studied the response of FPR2/ALX-transfected HL60 cells to culture filtrates of several important Gram-positive (*Streptococcus agalactiae*, *Enterococcus faecalis*, *Enterococcus faecium*) and Gram-negative (*Escherichia coli*, *Yersinia enterocolitica*, *Yersinia pseudotuberculosis*) pathogens. The CA-MRSA strains USA300 and USA400 were included as positive controls. In initial experiments, we measured calcium ion fluxes as a readout of leukocyte activation and chemotaxis. Untransfected HL60 cells showed no response even at the highest concentrations used (data not shown). FPR1-transfected HL60 responded in a dose-dependent fashion at relatively low rates, which were similar for all the tested strains except for *E. faecium*, which induced approximately two-fold higher calcium ion fluxes (Fig. 1A). These data are in agreement with the notion that all bacteria release formylated peptides at about similar levels [27], [28].

USA300 and USA400 induced very strong responses in FPR2/ALX-transfected HL60, which is in agreement with our previous findings [5]. Culture filtrates from *E. faecalis*, *E. coli*, *S. agalactiae*, *Y. enterocolitica*, and *Y. pseudotuberculosis* exhibited only weak FPR2/ALX-stimulating capacities, which did not exceed the FPR1-stimulating activities. However, *E. faecium* stimulated a strong response in FPR2/ALX-transfected HL60, which was much higher than in FPR1-transfected cells, but not as strong as the FPR2/ALX-dependent response to CA-MRSA strains (Fig. 1B).

### FPR2/ALX stimulation is a frequent trait in *E. faecium* but rare in *E. faecalis*

**Figure 2:**
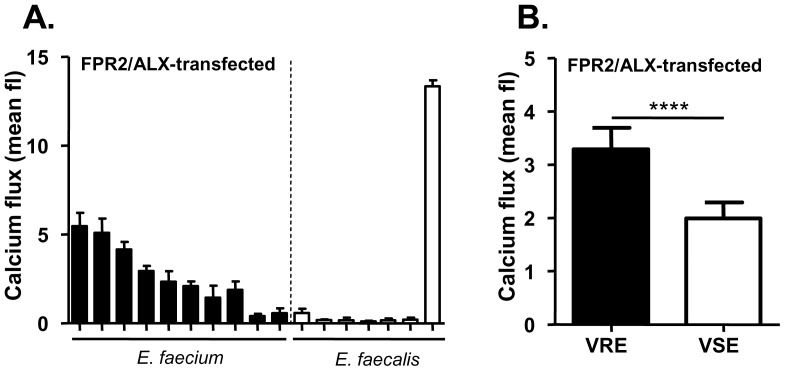
Calcium influx stimulated by enterococcal culture filtrates of various clinical blood culture or stool isolates. (A) Activation of FPR2/ALX-transfected cells by 3% *E. faecium* or *E. faecalis* culture filtrates. (B) Significantly stronger FPR2/ALX activation through 3% culture filtrates from vancomycin-resistant *E. faecium* (VRE) compared to vancomycin-sensitive *E. faecium* (VSE). Of each, VRE and VSE, five strains were analyzed. Data represent means ± SEM of 3 independent experiments and at least 3 different culture filtrates. P-value for Figure 2B was determined by the two-tailed Student's paired t-test. **** p<0.0001.

Ten *E. faecium* and eight *E. faecalis* strains, all clinical isolates from either stool samples or blood cultures, were compared for their FPR2/ALX-stimulating capacities to elucidate the prevalence of this property among clinical strains. Culture filtrates of eight of the *E. faecium* strains, of which three were vancomycin-resistant, exhibited substantial FPR2/ALX-specific activities, while in two other isolates the activities were weak (Fig. 2A). In contrast, the tested *E. faecalis* strains induced hardly any detectable activity in FPR2/ALX-transfected HL60 with only one exception. This *E. faecalis* strain, an isolate from a bacteremia patient, showed an extraordinarily high level of FPR2/ALX activation. Together, these findings suggest that FPR2/ALX activation may be rare among *E. faecalis* isolates, but very strong in certain strains.

There was no significant association of the FPR2/ALX-activating capacity of enterococcal strains with origin from intestinal colonization versus invasive infection. However, among the ten strains tested here vancomycin-resistant *E. faecium* exhibited significantly higher FPR2/ALX-stimulating properties than vancomycin-susceptible strains (Fig. 2B), suggesting that strong FPR2/ALX activation may be more frequent in health-care associated strains.

### *E. faecium* induces chemotaxis and complement receptor upregulation in human neutrophils in a FPR2/ALX-dependent manner

**Figure 3:**
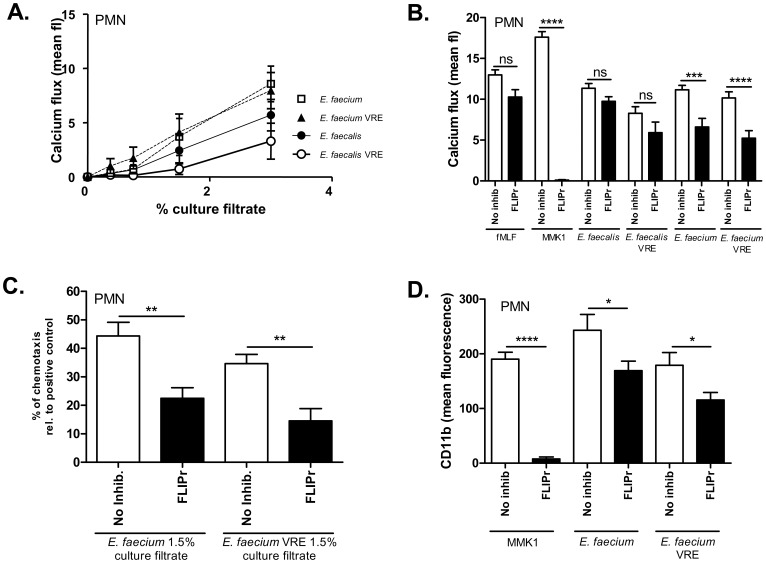
Enterococcal supernatants induce calcium influx and chemotaxis in human neutrophils. (A) Culture supernatants of *E. faecalis* and *E. faecium*, both vancomycin-resistant and vancomycin-sensitive, activate human leukocytes at different levels. (B) Activation of human leukocytes by *E. faecium* culture filtrates is stronger inhibited by the FPR2/ALX-specific inhibitor FLIPr than by *E. faecalis* culture filtrates. (C) Chemotaxis induced by *E. faecium* culture supernatants is inhibited by the FPR2/ALX-specific inhibitor FLIPr. (D) CD11b upregulation by *E. faecium* and *E. faecium* VRE 3% culture filtrates can be inhibited by FLIPr. Data represent means ± SEM of 3 independent experiments with 3 different culture filtrates. P-values were determined by one-way ANOVA with Bonferroni's post test. * p<0.05, ** p<0.001, *** p<0.0005, **** p<0.0001; ns, non-significant.

Culture filtrates of selected *E. faecium* and *E. faecalis* strains induced calcium fluxes in human neutrophils in a dose-dependent manner (Fig. 3A). In order to assess the role of FPR2/ALX in enterococcal neutrophil activation we used the FLIPr protein, which has been proven to be a highly specific inhibitor of FPR2/ALX [5], [7], [29]. FLIPr had no significant impact on the PMN-stimulating activity of a synthetic FPR1 ligand (fMLF) [7] but completely blocked the activity of a synthetic FPR2/ALX ligand peptide (MMK1) [7] (Fig. 3B) thereby confirming the specificity of FLIPr. The stimulating activities from *E. faecalis* could only slightly be inhibited by FLIPr (Fig. 3B), which is in agreement with the low FPR2/ALX-stimulating capacity in *E. faecalis* supernatants. Furthermore, this suggests that most of the neutrophil stimulation was mediated via FPR1. However, FLIPr had a stronger capacity to inhibit calcium fluxes stimulated by *E. faecium* culture filtrates in neutrophils, indicating that FPR2/ALX plays a more important role in neutrophil activation by *E. faecium* compared to *E. faecalis*. Similar results were obtained when neutrophil chemotaxis and CD11b upregulation in response to two selected *E. faecium* strains were studied (Fig. 3C, D).

### *E. faecium* FPR2/ALX agonists are proteinaceous without obvious similarity to staphylococcal PSMs

**Figure 4:**
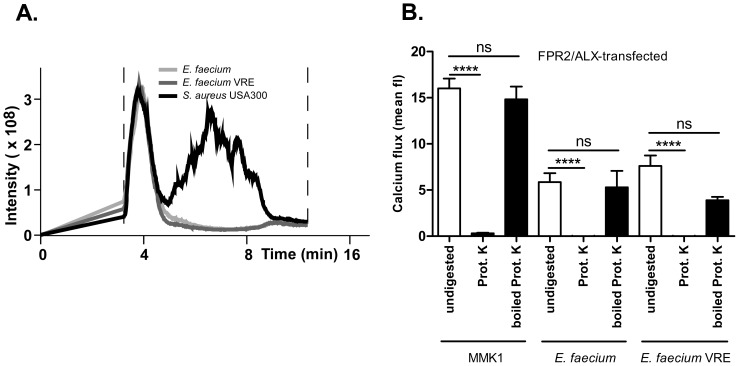
No detectable PSM-like peptides in *E. faecium* culture filtrates (A) and inactivation of FPR2/ALX-dependent activation by proteolytic treatment of culture filtrates (B). (A) 100 µl of 24-h culture filtrates of *E. faecium*, *E. faecium* VRE, and *S. aureus* USA300 were analyzed for the presence of PSMs using RP-HPLC/ESI-MS. The graph shows total ion chromatograms recorded in the characteristic PSM elution range (between dotted vertical lines). (B) Activation of FPR2/ALX-transfected cells is abolished by proteolytic digestion of *E. faecium* culture filtrates. Bacterial culture filtrate concentration was 1.5%. MMK1 concentration was 50 nM. Proteinase K was used at 1 unit ml^−1^. Data represent means ± SEM of 3 independent experiments with 3 different culture filtrates. P-values were determined by one-way ANOVA with Bonferroni's post test. * p<0.05, ** p<0.001, *** p<0.0005, **** p<0.0001; ns, non-significant.

While the chance to detect potential *psm-*related genes in bacterial genomes is limited because several PSM peptides show only low sequence similarities, at least the amino acid sequences of the PSMβ peptide class exhibit considerable conservation among different staphylococcal species [8]. Furthermore, the *psm*β genes are long enough to be included in genome open reading frame annotations. However, when we screened the available enterococcal genome sequences for genes with similarity to staphylococcal *psm* genes, we did not find any related sequences. Furthermore, when we analyzed culture filtrates from *E. faecalis* strains with FPR2/ALX-activating capacities that are in the range of those shown previously for some staphylococcal species with detectable PSM production [8], no peaks at PSM-characteristic elution times were detected using RP-HPLC/ESI-MS (Fig. 4A). Moreover, the capacity of enterococcal strains to activate FPR2/ALX did not correlate with synergistic hemolytic activity in the culture filtrates (data not shown), which is a typical property of staphylococcal PSMs [30], [31]. However, when *E. faecium* filtrates were treated with proteinase K, their capacities to stimulate FPR2/ALX-transfected HL60 were completely abolished. The inhibitory effect of proteinase K was not observed when the enzyme had been heat-inactivated prior to incubation with *E. faecium* and *E. faecium* VRE culture filtrates (Fig. 4B). Taken together, these data indicate that *E. faecium* produces proteinaceous ligands of FPR2/ALX and it appears that these ligands are not PSM-related.

## Discussion

While most of the known PAMP receptors sense molecules from major groups of bacteria or viruses, the FPR2/ALX receptor appears to respond only to a limited number of Gram-positive bacterial pathogens. Our study indicates that in addition to most staphylococcal pathogens and opportunists, FPR2/ALX can also detect molecules from *E. faecium* and selected *E. faecalis* clones. Staphylococci and enterococci are constituents of the skin and intestinal mammalian microflora, respectively, and together they are responsible for a majority of severe invasive human infections [10], [11], [32], [33]. It may be beneficial for the mammalian innate immune systems to use a leukocyte receptor that can specifically detect many of the most frequent bacterial pathogens and direct the recruitment and activation of neutrophils [34], [35].

The staphylococcal FPR2/ALX ligands, the PSM peptides, are important virulence factors and also have crucial roles in biofilm formation [5], [6], [36]. Therefore, FPR2/ALX has been proposed to sense not only the presence of invading microbes but also instruct the innate immune system about the virulence potential of the invaders [8]. It remains unclear if this notion also holds true for the enterococcal FPR2/ALX ligands. However, the fact that the source of the enterococcal strains from gut or from invasive infection and the level of synergistic hemolysis did not correlate with the capacity to stimulate FPR2/ALX suggests that the enterococcal FPR2/ALX ligands may not contribute much to enterococcal virulence. Nevertheless, the significant association of strong FPR2/ALX-agonistic activity with vancomycin resistance may indicate that health care-associated *E. faecium* have a stronger tendency to stimulate FPR2/ALX than community-associated *E. faecium*. We can only speculate about possible reasons for the large differences in the release of FPR2/ALX-stimulating activities among the various enterococcal strains. Expression of bacterial modulins such as PSMs is often controlled by regulatory mechanisms [26], [37] and certain modulin genes can be exchanged by horizontal gene transfer [38]. Thus, it is possible that expression of the enterococcal ligand proteins or peptides varies because of regulatory mutations or differences in the presence of mobile genetic elements.

In the present study, we have demonstrated that the *E. faecalis* FPR2/ALX agonists are proteinaceous, but their exact nature remains to be elucidated. FPR2/ALX responds to a variety of host or microbial peptides with unrelated sequences. The ligand signatures have remained largely elusive and are possibly defined by peptide secondary structure [5], [6]. Our attempts to identify potential agonist genes in the available *E. faecium* genomes failed, supporting our notion that the enterococcal FPR2/ALX ligands are no close homologs of PSM peptides. However, this does not exclude the possibility that *E. faecium* genomes contain *psm*-like genes, owing to the fact that also staphylococcal *psm* genes are frequently unrelated between species. The enterococcal ‘sex pheromone’ peptides cAM373 and cPD1 previously identified as chemotactic ligands for human leukocytes, are unrelated in sequences and lengths to PSMs and did not stimulate FPR2/ALX (data not shown). Thus, identification of the enterococcal FPR2/ALX agonists will require a comprehensive fractionation and activity testing approach.

The FPR2/ALX-activating capacities of enterococcal culture filtrates were considerably lower than those of CA-MRSA and the amounts required for inducing IL-8 and oxidative burst were much higher than those required to elicit calcium ion influx and chemotaxis (Fig. 3 and data not shown), which is in agreement with our recent finding that the amounts of FPR2/ALX ligands often differ between aggressive and opportunistic bacterial pathogens [8]. Since formyl-peptide receptor ligands can be active already at picomolar concentrations [4], [5], [24] and our observations suggest that the enterococcal FPR2/ALX ligands may be produced only at low levels, we expect identification of the chemical nature of enterococcal FPR2/ALX agonists to be extremely difficult.

Although many molecules, such as aggregation substance, cytolysin, capsule, sex pheromones, pili, glycolipids, or cell wall-anchored surface proteins, have been implicated in enterococcal virulence, how the innate immune system senses enterococci has remained incompletely understood [9], [39]. Our study indicates that there are proteinaceous mediators involved in *Enterococcus*-host interaction that await detailed characterization regarding chemical nature and role in virulence. Notably, our data underscore the importance of FPR2/ALX in Gram-positive bacterial infections and indicate that FPR2/ALX should be considered as a target for anti-infective and anti-inflammatory therapies.

## Acknowledgments

The authors would like to thank Nele Nikola and Monika Sinzel for excellent technical support, Kok van Kessel and Jos van Strijp for recombinant FLIPr, Sabine Gröbner for clinical enterococcal strains, and Francois Boulay for transfected cell lines.

## References

[1] Medzhitov R (2007). Recognition of microorganisms and activation of the immune response.. Nature.

[2] Ye RD, Boulay F, Wang JM, Dahlgren C, Gerard C (2009). International Union of Basic and Clinical Pharmacology. LXXIII. Nomenclature for the formyl peptide receptor (FPR) family.. Pharmacol Rev.

[3] Marasco WA, Phan SH, Krutzsch H, Showell HJ, Feltner DE (1984). Purification and identification of formyl-methionyl-leucyl-phenylalanine as the major peptide neutrophil chemotactic factor produced by Escherichia coli.. J Biol Chem.

[4] Migeotte I, Communi D, Parmentier M (2006). Formyl peptide receptors: a promiscuous subfamily of G protein-coupled receptors controlling immune responses.. Cytokine Growth Factor Rev.

[5] Kretschmer D, Gleske AK, Rautenberg M, Wang R, Koberle M (2010). Human formyl peptide receptor 2 senses highly pathogenic Staphylococcus aureus.. Cell Host Microbe.

[6] Wang R, Braughton KR, Kretschmer D, Bach TH, Queck SY (2007). Identification of novel cytolytic peptides as key virulence determinants for community-associated MRSA.. Nat Med.

[7] Prat C, Bestebroer J, de Haas CJ, van Strijp JA, van Kessel KP (2006). A new staphylococcal anti-inflammatory protein that antagonizes the formyl peptide receptor-like 1.. J Immunol.

[8] Rautenberg M, Joo HS, Otto M, Peschel A (2011). Neutrophil responses to staphylococcal pathogens and commensals via the formyl peptide receptor 2 relates to phenol-soluble modulin release and virulence.. FASEB J.

[9] Sava IG, Heikens E, Huebner J (2010). Pathogenesis and immunity in enterococcal infections.. Clin Microbiol Infect.

[10] Hidron AI, Edwards JR, Patel J, Horan TC, Sievert DM (2008). NHSN annual update: antimicrobial-resistant pathogens associated with healthcare-associated infections: annual summary of data reported to the National Healthcare Safety Network at the Centers for Disease Control and Prevention, 2006–2007.. Infect Control Hosp Epidemiol.

[11] Arias CA, Murray BE (2012). The rise of the Enterococcus: beyond vancomycin resistance.. Nat Rev Microbiol.

[12] Hirt H, Schlievert PM, Dunny GM (2002). In vivo induction of virulence and antibiotic resistance transfer in Enterococcus faecalis mediated by the sex pheromone-sensing system of pCF10.. Infect Immun.

[13] Chow JW, Thal LA, Perri MB, Vazquez JA, Donabedian SM (1993). Plasmid-associated hemolysin and aggregation substance production contribute to virulence in experimental enterococcal endocarditis.. Antimicrob Agents Chemother.

[14] Schlievert PM, Gahr PJ, Assimacopoulos AP, Dinges MM, Stoehr JA (1998). Aggregation and binding substances enhance pathogenicity in rabbit models of Enterococcus faecalis endocarditis.. Infect Immun.

[15] Sannomiya P, Craig RA, Clewell DB, Suzuki A, Fujino M (1990). Characterization of a class of nonformylated Enterococcus faecalis-derived neutrophil chemotactic peptides: the sex pheromones.. Proc Natl Acad Sci U S A.

[16] Ember JA, Hugli TE (1989). Characterization of the human neutrophil response to sex pheromones from Streptococcus faecalis.. Am J Pathol.

[17] Wirth R (1994). The sex pheromone system of Enterococcus faecalis. More than just a plasmid-collection mechanism?. Eur J Biochem.

[18] Schulte B, Heininger A, Autenrieth IB, Wolz C (2008). Emergence of increasing linezolid-resistance in enterococci in a post-outbreak situation with vancomycin-resistant Enterococcus faecium.. Epidemiol Infect.

[19] Christophe T, Karlsson A, Dugave C, Rabiet MJ, Boulay F (2001). The synthetic peptide Trp-Lys-Tyr-Met-Val-Met-NH2 specifically activates neutrophils through FPRL1/lipoxin A4 receptors and is an agonist for the orphan monocyte-expressed chemoattractant receptor FPRL2.. J Biol Chem.

[20] Dahlgren C, Christophe T, Boulay F, Madianos PN, Rabiet MJ (2000). The synthetic chemoattractant Trp-Lys-Tyr-Met-Val-DMet activates neutrophils preferentially through the lipoxin A(4) receptor.. Blood.

[21] Durr MC, Kristian SA, Otto M, Matteoli G, Margolis PS (2006). Neutrophil chemotaxis by pathogen-associated molecular patterns–formylated peptides are crucial but not the sole neutrophil attractants produced by Staphylococcus aureus.. Cell Microbiol.

[22] Marasco WA, Phan SH, Krutzsch H, Showell HJ, Feltner DE (1984). Purification and identification of formyl-methionyl-leucyl-phenylalanine as the major peptide neutrophil chemotactic factor produced by Escherichia coli.. J Biol Chem.

[23] Fu H, Karlsson J, Bylund J, Movitz C, Karlsson A (2006). Ligand recognition and activation of formyl peptide receptors in neutrophils.. J Leukoc Biol.

[24] Le Y, Murphy PM, Wang JM (2002). Formyl-peptide receptors revisited.. Trends Immunol.

[25] Nanamori M, Cheng X, Mei J, Sang H, Xuan Y (2004). A novel nonpeptide ligand for formyl peptide receptor-like 1.. Mol Pharmacol.

[26] Kretschmer D, Nikola N, Durr M, Otto M, Peschel A (2012). The virulence regulator Agr controls the staphylococcal capacity to activate human neutrophils via the formyl peptide receptor 2.. J Innate Immun.

[27] Schiffmann E, Corcoran BA, Wahl SM (1975). N-formylmethionyl peptides as chemoattractants for leucocytes.. Proc Natl Acad Sci U S A.

[28] Mader D, Rabiet MJ, Boulay F, Peschel A (2010). Formyl peptide receptor-mediated proinflammatory consequences of peptide deformylase inhibition in Staphylococcus aureus.. Microbes Infect.

[29] Prat C, Haas PJ, Bestebroer J, de Haas CJ, van Strijp JA (2009). A homolog of formyl peptide receptor-like 1 (FPRL1) inhibitor from Staphylococcus aureus (FPRL1 inhibitory protein) that inhibits FPRL1 and FPR.. J Immunol.

[30] Wiseman GM (1975). The hemolysins of Staphylococcus aureus.. Bacteriol Rev.

[31] Cheung GY, Duong AC, Otto M (2012). Direct and synergistic hemolysis caused by Staphylococcus phenol-soluble modulins: implications for diagnosis and pathogenesis.. Microbes Infect.

[32] Passerini R, Ghezzi T, Sandri M, Radice D, Biffi R (2011). Ten-year surveillance of nosocomial bloodstream infections: trends of aetiology and antimicrobial resistance in a comprehensive cancer centre.. Ecancermedicalscience.

[33] Wisplinghoff H, Bischoff T, Tallent SM, Seifert H, Wenzel RP (2004). Nosocomial bloodstream infections in US hospitals: analysis of 24,179 cases from a prospective nationwide surveillance study.. Clin Infect Dis.

[34] Holtfreter S, Jursa-Kulesza J, Masiuk H, Verkaik NJ, de Vogel C (2011). Antibody responses in furunculosis patients vaccinated with autologous formalin-killed Staphylococcus aureus.. Eur J Clin Microbiol Infect Dis.

[35] Verkaik NJ, Dauwalder O, Antri K, Boubekri I, de Vogel CP (2010). Immunogenicity of toxins during Staphylococcus aureus infection.. Clin Infect Dis.

[36] Periasamy S, Joo HS, Duong AC, Bach TH, Tan VY (2012). How Staphylococcus aureus biofilms develop their characteristic structure.. Proc Natl Acad Sci U S A.

[37] Queck SY, Jameson-Lee M, Villaruz AE, Bach TH, Khan BA (2008). RNAIII-independent target gene control by the agr quorum-sensing system: insight into the evolution of virulence regulation in Staphylococcus aureus.. Mol Cell.

[38] Queck SY, Khan BA, Wang R, Bach TH, Kretschmer D (2009). Mobile genetic element-encoded cytolysin connects virulence to methicillin resistance in MRSA.. PLoS Pathog.

[39] Clewell DB, Clewell DB (1993). Sex pheromones and the plasmid-encoded mating response in Enterococcus faecalis..

[40] Sahm DF, Kissinger J, Gilmore MS, Murray PR, Mulder R (1989). In vitro susceptibility studies of vancomycin-resistant Enterococcus faecalis.. Antimicrob Agents Chemother.

[41] Diep BA, Gill SR, Chang RF, Phan TH, Chen JH (2006). Complete genome sequence of USA300, an epidemic clone of community-acquired meticillin-resistant Staphylococcus aureus.. Lancet.

[42] Baba T, Takeuchi F, Kuroda M, Yuzawa H, Aoki K (2002). Genome and virulence determinants of high virulence community-acquired MRSA.. Lancet.

[43] Werth N, Beerlage C, Rosenberger C, Yazdi AS, Edelmann M (2010). Activation of hypoxia inducible factor 1 is a general phenomenon in infections with human pathogens.. PLoS One.

[44] Heesemann J, Laufs R (1983). Construction of a mobilizable Yersinia enterocolitica virulence plasmid.. J Bacteriol.

[45] Tsubokura M, Aleksic S, Fukushima H, Schulze G, Someya K (1993). Characterization of Yersinia pseudotuberculosis serogroups O9, O10 and O11; subdivision of O1 serogroup into O1a, O1b, and O1c subgroups.. Zentralbl Bakteriol.

